# Understanding and accounting for relational context is critical for social neuroscience

**DOI:** 10.3389/fnhum.2014.00127

**Published:** 2014-03-25

**Authors:** Elizabeth Clark-Polner, Margaret S. Clark

**Affiliations:** ^1^Department of Psychology, University of ChicagoChicago, IL, USA; ^2^Department of Psychology, Trumbull College, Yale UniversityNew Haven, CT, USA

**Keywords:** relational context, attachment styles, relationship histories, relationship types, relationship character, relationship stages

## Abstract

Scientists have increasingly turned to the brain and to neuroscience more generally to further an understanding of social and emotional judgments and behavior. Yet, many neuroscientists (certainly not all) do not consider the role of relational context. Moreover, most have not examined the impact of relational context in a manner that takes advantage of conceptual and empirical advances in relationship science. Here we emphasize that: (1) all social behavior takes place, by definition, within the context of a relationship (even if that relationship is a new one with a stranger), and (2) relational context shapes not only social thoughts, feelings, and behaviors, but also some seemingly non-social thoughts, feelings, and behaviors in profound ways. We define relational context and suggest that accounting for it in the design and interpretation of neuroscience research is essential to the development of a coherent, generalizable neuroscience of social behavior. We make our case in two ways: (a) we describe some existing neuroscience research in three substantive areas (perceiving and reacting to others’ emotions, providing help, and receiving help) that already has documented the powerful impact of relational context. (b) We describe some other neuroscience research from these same areas that has *not* taken relational context into account. Then, using findings from social and personality psychology, we make a case that different results almost certainly would have been found had the research been conducted in a different relational context. We neither attempt to review all evidence that relational context shapes neuroscience findings nor to put forward a theoretical analysis of all the ways relational context ought to shape neuroscience findings. Our goal is simply to urge greater and more systematic consideration of relational context in neuroscientific research.

## INTRODUCTION

What makes a thought, feeling, or behavior “social”? A reasonable criterion is that the thought, feeling, or behavior is social if it arises from an individual’s interdependence with another person. In other words, a person’s thoughts, feelings, and/or behaviors can be considered “social” if they influence and/or are influenced by another person’s thoughts, feelings, and/or behavior. This definition highlights an important point: social acts cannot, by definition, be understood by focusing on one actor alone; they must be examined as *multi*-person processes – processes that are powerfully shaped by the nature of the interdependence that exists, or is desired, between persons – or, in other words, by what we here call relational context.

The study of social behavior using *any* methodology, including neuroscience methodologies, must take relational context into account. Researchers must consider not only the nature of the actor him or herself, but also the nature of the person with whom he or she is interacting, and, crucially, *the nature of the existing (or desired) relationship between them*. It is this last aspect of multi-person processes on which we focus in this paper.

Whereas large literatures have accumulated in which researchers explain interpersonal interactions in terms of the characteristics of the actor (the participant in a research study), or, alternatively, the characteristics of person with whom he or she is interacting (the target person), far less frequently do researchers account for, or attempt to explain behavior in terms of the characteristics of the relationship *between* the actor and the target. These characteristics are, however, patently important: many crucial social variables inhere primarily in relational ties (or perceived relational ties), rather than in individuals or in targets themselves. Variables such as interpersonal similarity, trust, commitment, empathy, hostility, felt obligation, and prosocial behavior make no sense outside the context of a relationship. Moreover, most variability in such contacts occurs between the different relationships within individuals’ sets of relationships, rather than between individuals themselves. (We hasten to acknowledge, of course, that chronic differences in peoples tendencies to feel such things as trust, commitment or empathy, and to elicit them from others do exist. Yet even these differences are inherently relational in nature, in that they are best understood as having developed in the context of specific relationships, and then continuing to have their impact in similar relationships.)

Failing to account for relational context in our research, we suggest, has consequences beyond simply precluding researchers from maximizing their knowledge about social behavior. By collectively neglecting relational context as a variable of interest, while letting it vary across studies, researchers risk producing a confusing literature. Furthermore, by ignoring relational context in their empirical work, individual researchers risk drawing conclusions that will have limited generalizability.

Many current theoretical and empirical neuroscience models of social behavior have been developed largely upon the basis of research in which the participants are interacting with, or acting in the presence of, other individuals whom they have not met before and likely never expect to see again (e.g., Sanfey et al., 2008). These models may or may not be valid when it comes to predicting the actions and interactions within the context of established relationships – which are among the most common, and consequential, actions and interactions people, execute every day. To be fair, a growing amount of social neuroscience research has been conducted in the context of ongoing relationships. Moreover, some has involved participants actually interacting with one another as neuroscientific measures have been collected. Yet often, even work such as this is not accompanied with a careful conceptual consideration of the nature of the relational context involved, nor does it involve intentional, experimental manipulation of relational contexts within studies, or even comparison of results collected within different relational contexts. Conceptual analyses and consideration of relational contexts, as well as studies involving manipulation of relational contexts and comparison of results across distinct studies that have addressed the same questions in differing relational contexts, are all necessary to allow the researchers to determine how relational contexts shape psychological processes.

In what follows, we make a case for greater consideration of relational context in neuroscience research. We begin with a brief overview of some ways in which relational context influences social behavior. We then illustrate our point – that relational context matters for social neuroscience – with empirical examples drawn both from the social neuroscience and behavioral literatures. Broadly speaking, we posit that: (a) much neuroscientific research (and much behavioral research as well) neglects relational context; (b) the large behavioral literatures on topics listed above provide strong evidence that relational context matters, and (c) that neuroscientists who have taken relational context into account have provided us with additional strong evidence that relational context matters.

We have chosen to focus primarily on research in three domains of social behavior to illustrate our points: (a) the expression and perception of emotion, (b) giving social support, and (c) receiving social support. In each case, the point we make is simple: relational context matters. We believe, however, that the importance of relational context goes far beyond these three domains and that it is influential not only with regard to many other social behaviors (e.g., attitude formation and change, conformity, prejudice and stereotyping, etc.), but also with regard to some seemingly non-social domains as well such as perception (Schnall et al., 2008) and intelligence (Woolley et al., 2010). We elaborate on the latter point a bit below.

## RELATIONAL CONTEXT

As stated above, all social behaviors are, by definition, interpersonal (as the saying goes, it takes two to tango!”). Anything that is interpersonal, furthermore, takes place within the context of a *relationship*. This is true even if that interaction is between people who have never met before, will never meet again, and who have no acquaintances in common – this is simply *one type* of relationship (one that exists between strangers who expect to remain so). This does not mean that interactions can only occur between two individuals (they could take place, for example, between an individual and a group, or between two groups), but rather that they *cannot* occur within one person, in isolation. The other person may be (and typically is) present but need not be; for instance, one can act in such a manner so as to benefit a person who is not present. The point remains that the study of social behavior must take into account not only the actor, but also the person with whom she or he is interacting, and the nature of the existing, desired or past relationship between them.

The relationship aspect of this equation is particularly important because the nature of the relationship that exists between people is a key determinant of the norms governing interactions, how partner behavior will be interpreted, what duties are felt toward the partner, how much attention will be paid to the partner and the list could go on. Relational context influences not only if, when and how people will act socially toward one another, and how they will respond to that other’s behavior but, importantly, it also defines what counts as “pro-,” “anti-,” or neutral social behavior.

Consider a simple example: what happens when a beautiful bouquet of flowers is delivered to a woman’s home? How will she react? It depends on relational context. If the flowers come from a suitor to whom she is attracted, and if she has been hoping the attraction is mutual, acceptance of the flowers and joy will result. If they come from her spouse of 30 years who has sent flowers every week for all those years, she will accept the flowers but may have no emotional reaction. If they come from a suitor who is nice enough but in whom this woman personally has no interest, reluctance to accept them, distress and perhaps feelings of guilt may arise. If they come from a person who has been stalking the woman and against whom she has a protective order, she will refuse the flowers and feel fear and distress. The point is straightforward: behavior, cognition, and emotion depend on relational context.

Before we can examine how relational context influences social behavior in more detail, however, it will be useful to understand six ways in which relational context can vary.

### SIX WAYS IN WHICH RELATIONAL CONTEXT VARIES

When relational context has been noted in recent neuroscientific research, it is often referred to in terms of relationship types, with those types labeled in lay terms. Researchers have examined, for example, mother–child relationships and romantic relationships (e.g., Ortigue et al., 2010). Relational context certainly *can* be defined in lay language, yet that leaves open the questions of just how these relationships differ, *conceptually*, from one another, whether there is meaningful variation within a group of relationships labeled with the same term, and how relationships given different names overlap with one another in conceptual ways. We believe understanding relationships in conceptual terms to be crucial to the study of social life generally, and to social neuroscience in particular (Clark et al., in press). We specifically suggest that social and affective neuroscience (and, indeed, many areas of psychology) will benefit by first considering six ways in which relational context varies, and then considering conceptual variation within each. (Often researchers will be able to capture the same conceptual variation in different methodological ways if they explicitly consider all six ways in which relational context varies. For instance, trust of partners will vary both within an individual according to relationship type, between individuals’ in terms of chronic tendencies to trust other people, and within one individual’s specific relationship with another as that relationship develops.)

The ways in which relational context vary are: (1) the type of relationship; (2) the character (or “personality”) of the relationship (as distinct from the personalities of individuals involved in the relationship); (3) chronic individual differences in members’ orientations toward relationships; (4) the history of the relationships; (5) the developmental stage of the relationships; and (6) the broader relationship network within which a particular relationship being studied is embedded (Clark et al., in press). In what follows we provide a short description of each way of thinking about relational context.

#### Relationship type

As noted above people (including many researchers) tend to think of relationship types in lay terms. They talk about, for example, their friendships, romantic relationships, parent–child relationships, and work relationships. Yet relationship types have been defined differently within the social psychological literature that deals specifically with studying and characterizing these different types of interactions. One way relationships are commonly characterized by relationship scientists is in terms of the norms, implicit and explicit, that govern interactions with others in those relationships (Clark and Mills, 1979, 1993, 2012; Fiske, 1992). These norms arise from the social function(s) people play in one another’s lives (Bugental, 2000; Clark and Mills, 2012). Alternatively these can be conceptualized as the goals that people pursue, ultimately or proximately, in a given relationship.

In many cases, the goals that people pursue in given relationships and the social functions that relationships serve in their lives do differ along traditional lay-defined relationship lines. Friendship, for example, often serves the function of providing both members with a sense of security based on each member following an implicit rule to provide the other with non-contingent support aimed at maintaining and promoting the other’s welfare. A romantic relationship may serve this same function but, importantly, it serves another function as well, providing for sexual gratification. So too does it serve the (ultimate) function of preserving genes by helping people reproduce and to raise children to the point of sexual maturity and reproduction themselves (see Bugental, 2000). Thus, lay terms do capture some important variance in relationship type, *but* lay language does not make it clear just what is being captured, conceptually. If researchers think more in terms of social functions of relationships and of how those functions manifest themselves, they will be better off scientifically.

For example, as just stated, people rely on some relationship partners to be non-contingently responsive to their needs. These relationships (known as communal relationships) provide people with a sense of security and flexibility in seeking as well as giving support. Friendships, romantic relationships, and family relationships often (but not always!) exemplify communal relationships. In other relationships people do not assume nor desire such non-contingent responsiveness to needs and desires. Yet they may still wish to seek and give support to partners in a different, less committing may. Imagine that a person’s drains are clogged and that person seeks a plumber’s assistance. In such a case the person may wish to form what has been called an exchange relationship (Clark and Mills, 1979, 1993; Clark and Aragon, 2013) in which that person can seek support and provide compensation. In exchange relationships, individuals provide benefits to each other with the expectation that these actions will be repaid; the benefits are given contingently, and the individuals feel no particular non-contingent and ongoing responsibility for each other’s welfare, beyond that which they feel for any other person (Clark and Mills, 1979; Mills and Clark, 1982).

The communal/exchange distinction, however, is just one conceptualization of relational context among many that may prove useful to neuroscientists. Other typologies capturing distinct social functions – ones that categorize relationships in terms of power or authority differences, in terms of sexual orientation, in terms of genetic relatedness, for example – will prove useful for different purposes. Our point is not to cover them all but just to urge researchers to think more in conceptual terms about relationship types and less in lay language terms, and to use the extant literature on relationships in so doing. Although there currently is no one scientific typology of relationship types that will adequately serve all research purposes (the research questions must guide the selection or generation of useful conceptualizations), we refer readers to Bugental (2000) for an example of a particularly clearly laid out typology regarding the various social functions a relationship may serve. She distinguishes relationships in terms of those which serve to keep us safe, those that allow collective acquisition and defense of resources and territories, those that promote mating, those involving reciprocity to maximize joint outcomes and those that allow us to optimize welfare by unequal distribution of power. She also discusses what sorts of information, neuro-hormonal regulators and social–emotional responses are relevant to each, as well as issues of development relating to each.

When thinking about relationship types, it is important to keep in mind that for purposes of conducting empirical work they can be captured both in terms of the distinct natures of existing, ongoing relationships as some researchers have done (e.g., Ortigue et al., 2010) but also that enacted, expected, and/or desired relationship types can effectively experimentally manipulated [see, for instance, Clark (1986) for a description of an experimental manipulation that effectively varies whether participants desire a communal or exchange relationship with a target person or De Bruijn and von Rhein (2012), Koban et al. (2010), and Radke et al. (2011) for other examples of effective experimental manipulations of relationship type]. Each approach has advantages and disadvantages. Use of existing relationships captures naturally occurring differences between relationships but often lacks control and leaves room for alternative explanations of observations. Conducting true experiments in which expected or desired relationship types are manipulated provides for more control but likely will not be feasible for studies of relationship types that take days, months, or years to develop.

Regardless of what strategy is used, relationship type is likely to account for a great deal of variability in how we express and perceive emotions, how and when we give and accept support and empathize with others, and in how we interact with others in economic or strategic situations. Even so, there is more to relational context beyond just these distinctions.

#### Relational character

To truly capture relational context one must also account for what amounts to the personality of a relationship, or *relational character*. Just as there are many aspects of an individual’s personality, so too are there many aspects of relational character that vary across, as well as within, relational types.

Take, for instance, communal relationships, which we described above. Clark and Mills (1979) identified communal relationships as those relationships in which partners provide benefits non-contingently, in support of one another’s welfare. Within this general category, however, relationships can vary in terms of communal strength, or, in other words, in the degree to which one assumes responsibility for the other’s welfare (Mills and Clark, 1982; Mills et al., 2004; Clark and Mills, 2012). Communal strength can be indexed by the effort, time, and cost one is willing to expend, in the service of (non-contingently) promoting the partner’s welfare. Communal strength also can be indexed by the relative priority one assigns to caring for a specific partner when one has multiple, communal relationships, the demands of which may conflict. This aspect of relationship character is central to determining the giving of support and feelings of guilt and to determining levels of distress when support is not given.

Communal strength, however, is just one of many aspects of relational character to which neuroscientists might profitably attend in their studies of social and emotional phenomena. Interdependence theorists, for instance, have discussed variation between relationships in terms of the degree to which people’s routines of thoughts, emotions and behaviors are dependent upon those of their partner. They index that degree of interdependence by how frequently, strongly, and in how many distinct ways members of the relationship influence each other’s routines (Kelley, 1979; Berscheid et al., 1989). Other examples of relational character include two persons’ *similarity* (along any of many possible dimensions; Amodio and Showers, 2005), the *trust* that exists between them (Simpson, 2007), the *certainty* that each person has regarding the existence and nature of their relationship (Clark et al., 1998), their *commitment* to remaining together (Rusbult, 1983), and the degree to which the partners are *satisfied* with the relationship (Hendrick, 1988). These dimensions of relational character overlap somewhat empirically as well as conceptually. There are not right or wrong ways in which to characterize relational character. What is important is to consider relational character in conducting social neuroscience work and to consider how it may shape whatever aspects of affect, cognition, and/or behavior are being studied.

As with relationship types, it is important for researchers to keep in mind that relational character can be measured and that it also can be effectively manipulated in many cases. We refer readers to Lamm et al. (2009) for a neuroscientific study in which the similarity of participants’ pain experiences to those of a target person was effectively varied (and in which similarity did have significant effects on participants’ empathic reactions to targets).

#### Individual differences in approaches to relationships

The third aspect of relational context is the chronic nature of individuals’ orientation toward their relationships *in general*. People are known to vary in prosocial orientation (e.g., Batson and Shaw, 1991; Grant and Mayer, 2009), in chronic levels of relationship insecurity captured by anxious and avoidant attachment styles (Mikulincer and Shaver, 2007), in rejection sensitivity (Downey and Feldman, 1996), in empathic self-efficacy beliefs (Alessandri et al., 2009), in communal orientation (Clark et al., 1987), in self-esteem (Leary and Downs, 1995) and the list could continue. These individual differences (many of which are interrelated though, to date, no one has documented fully the extent to which they overlap and/or are independent of one another) manifest themselves in the ways people relate to more than one relationship, perhaps to all social relationships or to all social relationships within a category of relationship types. These individual differences also may interact with situational factors, including relationship types, to determine attention to others’ feelings, needs and desires, and reactions to receiving support.

Individual differences also may influence a person’s ability to effectively form and carry out the functions of any given relationship type. For instance, a person characterized by avoidant attachment may have difficulty forming and carrying out the functions of a communal relationship (see Simpson et al., 1992 for a particularly clear and compelling illustration of this point). This highlights another reason for researchers to be cautious regarding using lay language terms to characterize relationship types. All people may say they have friends, for instance. Yet not all relationships called friendships will be characterized by members feeling secure in partner responsiveness and being able to effectively give and receive responsiveness. In other words, not all relationships called friendships can be assumed to be the same conceptually. The very nature of “friendships” will almost certainly differ with the individual differences that people bring with them to those relationships (see Clark and Lemay, 2010 for a full description of this).

These individual differences constitute a part of what we mean by relational context, and constitute a distinct way of considering relational context both from relationship type and from the relational character of specific relationships. Still, in many cases, the individual differences that people bring to relationships will blend and interact with relationship type and relationship character to predict psychological reactions and processes^1^.

#### Relationship histories and anticipated relationship futures

Existing relationships also have histories that must be taken into account. A relationship’s history plays a role in shaping both partners’ behavior, their perceptions of events, and their reactions to those events. History with a partner creates expectations, and these expectations influence how one behaves toward a partner (Baldwin, 1992). An established pattern of interdependence with a partner, for instance, leads to firm expectations for future behavior, and can set the stage for feelings of emotions (both positive and negative) when those expectations are broken (Berscheid and Ammazzalorso, 2001).

Relationship history with one partner can also influence thoughts, feelings, and behavior in a different relationship (Coan et al., 2013a), especially if a current partner reminds a person in some way of a past partner (Chen et al., 2013). Relationship histories that influence new relationships more generally may sometimes be best conceptualized as individual differences in approaches to relationships (our category #3 here).

The anticipated *future* of a relationship also can shape social desires, emotional judgments, and behavior in the present (cf. Clark and Mills, 1979). Receiving a gift from someone with whom one anticipates forming a friendship or romantic relationship, for example, will elicit a different response than receiving the same gift from someone whom, one is certain, one will not be seeing again in the future.

#### Developmental stage of relationships

The developmental stage of a relationship also may have important consequences when it comes to social behavior^2^. Relationships have a time course that interacts with their functions and goals. All relationships change over time (Mitnick et al., 2009). Friendships or business relationships between peers, for example, will have establishment, maintenance, and – perhaps – deterioration stages. Another (overlapping) way of thinking about relationship stages is in terms of there being a deliberative stage of a relationship (involving deciding whether one wishes to be in the relationship and what type of relationship one desires) and an implemental stage (involving implementing the appropriate behaviors within an established relationship type; cf. Gollwitzer et al., 1990; Gagne and Lydon, 2001a,b). The stage of a relationship is an important predictor of the social and emotional processes that will occur in that relationship (Gagne and Lydon, 2001a,b; Beck and Clark, 2010; Clark and Beck, 2011). Social and affective reactions to others change across time as relationships unfold.

#### Placement of a relationship within wider relationship networks

The last aspect of relational context worthy of mention concerns the placement of a particular relationship within each person’s larger set of relationships – i.e., in their social *network*. Just as individual interpersonal interactions occur within the context of specific relationships, so too do relationships function within the context of a person’s larger social network, and this also will influence social and emotional behavior. For instance, attention to, and favorable judgments of, the physical attractiveness of potential romantic relationship partners have been shown to be decreased by the existence of, and commitment to, an existing romantic relationship partner (Johnson and Rusbult, 1989) and this moderation is manifest even in very fast, automatic, and non-conscious processes (Maner et al., 2008). A familiar face should be more comforting when spotted in the context of many unfamiliar faces in a new social situation (in which people tend to be anxious) than when seen in the context of many other familiar faces in established social situations in which people are happy and comfortable (cf. Vanbeselaere, 1980; Mikulincer et al., 2002; DeVries et al., 2010). A person who is perfectly comfortable with a friend when they are alone as a pair may be embarrassed by being associated with that same friend when in the company of additional peers (cf. Fortune and Newby-Clark, 2008).

In sum, relational context is complex. It includes relationship type, relationship character, individual differences in orientations toward relationships, relational history and stage, and the placement of a given relationship in the wider context of a person’s other relationships. No matter what a researcher’s substantive interests, we believe it will be useful for that researcher to consider all these aspects of relational context. A person interested in empathy as a process, for instance, may wish to consider variation in empathic processes between types of relationships, within types of relationships, at different points in a relationship’s history and stage as well as how empathy is influenced by the wider social network into which a relationship fits. We have little doubt that an explicit consideration of conceptual variables as they are captured in each type of relational context will prove to be useful and, when taken into account in planning and conducting research, will make it easier to integrate findings, both within neuroscience, and across neuroscience and relationship science more broadly.

## THE IMPORTANCE OF CONSIDERING SOCIAL NEUROSCIENCE FINDINGS IN RELATIONAL CONTEXT

Here we have already argued that (1) all social behavior takes place, by definition, within the context of relationships, and that (2) relational context often affects the nature of results obtained in neuroscientific and other studies of psychological processes. In order to illustrate these points, we have chosen to discuss, as examples, a few specific types of neuroscience research on a few measures of social behavior. First, we examine relational context and the expression and perception of emotion. Next we look at the impact it has on empathy, and then we consider the impact it has on the giving and receiving of social support. Finally, we will briefly comment on how relational context influences even some seemingly non-social thoughts, feelings and behaviors. In each case, the point we will make is simple: relational context matters. Specifically, we suggest that: (a) much neuroscientific research has ignored relational context; (b) the largely separate behavioral literature in this area provides strong evidence that relational context matters; and (c) those neuroscience studies that *have* taken relational context into account generally show that relational context does matter.

### PERCEIVING AND REACTING TO OTHERS’ EMOTIONS

There is now a large literature on the neural correlates of emotion perception. Within this literature, there is growing variation in the paradigms used (see discussions in, for example, Barrett, 2006; Scherer et al., 2011). Of particular importance is the fact that, in the vast majority of cases, the stimuli utilized in emotion perception paradigms have been depictions of people who are strangers to the participants (see Ebner et al., 2012; Montoya et al., 2012 for some recent examples but many more exist). Moreover often these are strangers whom participants actually are not meeting and whom they never expect to see again. This is the case in studies of reactions to facial (e.g., Xaoyun et al., 2009), vocal (e.g., Baum and Nowicki, 1998), and bodily (e.g., Coulson, 2004) expressions of emotion, and in cross-cultural (e.g., Yik et al., 1998), developmental (e.g., Camras et al., 2002), and clinical (e.g., Anderson and Phelps, 2000) research as well. This is troubling, because the broader psychological literature on relationships (as well as a growing amount of the neuroscience literature) provides good reason to believe that relational context has a major impact on how people perceive and react to others’ emotions.

#### Perceiving emotion: some findings that may not extend beyond perceiving emotions in strangers whom one will never see again

When people encounter *strangers*, the primary function that emotion perception may serve is to protect or promote the self’s well-being by, say, avoiding angry people, approaching smiling people, and looking around to detect problems and to protect the self when someone else seems fearful (Klinnert et al., 1986). However, the functions that others’ emotional expressions serve become more complex when relational context is considered. When people know one another, when they are interdependent, and when they assume responsibility for one another’s welfare, others’ facial expressions continue to be signals that can be used to protect or promote the self, but they take on additional functions as well – one important one being that they serve as signals of the other person’s welfare and, as such, as signs that the perceiver should provide care to the other person. If a person is fearful perhaps one might reassure the other person, help the person distance him or herself from the feared stimulus, or remove the feared stimulus from the environment. If a person is happy, one might ask why and celebrate with the person, thereby prolonging the person’s happiness, leave the person alone to continue enjoying whatever is causing the happiness, or repeat one’s own actions if they were the source of the happiness as is appropriate to the situation.

For example, consider the implications of taking account of whether there is an existing caring (or communal) relationship – or the desire to establish a relationship – between a perceiver and an emotional target person for interpreting one recent study of reactions to others’ facial expressions. N’Diaye et al. (2009) had 24 individuals look at target faces expressing happiness, fear, or anger, in either mild or intense forms. They also varied the direction in which the target face was gazing, such that the person was either looking directly at the perceiver, or off to the side. Functional magnetic resonance imaging (fMRI) scans were collected. The researchers predicted that, for the faces expressing fear, the perceived self-relevance of the expression would be higher when the gaze was averted (which suggests that there is something in the environment for the self to fear) rather than when it was direct (suggesting that the person feared the participant), but that, for faces expressing anger, the perceived self-relevance would be greater when the target face was looking directly at the participant (which suggests that the emotional target is angry at the perceiver) than when it was averted. These predictions were supported. Behaviorally, ratings of emotion intensity were greater when fear was expressed with an averted gaze than with a direct gaze, and when anger was expressed with a direct gaze than with an averted gaze. The same pattern was reflected in terms of the neural correlates of emotion perception, in the amygdala, as well as in the fusiform and medial prefrontal cortices.

These results are intriguing, and the explanations makes good sense *given* that the target persons were strangers to the participants, which, in this study, they were. We watch out for and protect *ourselves* when with strangers. However, what if the target person had been a friend, a romantic partner, or a family member? What if he or she were simply someone attractive, with whom a participant desired a relationship? Then, viewers’ reactions to the emotions expressed, and the interaction effect with gaze direction that has been observed, we suggest, almost certainly would have been different.

Importantly, those who willingly express emotions to us are choosing to convey rather than to suppress information about their own well being (Clark et al., 2001). They are often in need of our support and, if we have assumed responsibility for their welfare (or wish to do so going forward), people often switch their relational focus of attention from themselves and the implications of others for themselves to the partner and what they can do for that partner (Clark et al., 2008). Perceivers then respond to others’ negative emotions with care (Clark et al., 1987; Graham et al., 2008). To give an obvious example, parents typically respond to an infant’s cries by shifting attention to the child, focusing on the child’s needs and providing care. Spouses and friends would likely do the same.

When a fearful face looking right at us belongs to a person for whom we have assumed or would *like* to assume responsibility, that should trigger a shift in relational focus of attention (Clark et al., 2008) *from* the self *to* the other person. The face then becomes an implicit request for help, *especially* in the direct gaze condition. Our reactions ought to be just as intense (or perhaps more intense), and likely different in nature both from those captured when looking at a fearful stranger, and from those captured when the person is gazing somewhere else (then, just as in the original study, we may react on our behalf as well as on their behalf). And what about angry gazes? In an intimate relationship in which another’s emotion signals us that our partner has a need to which we should attend, an angry person’s averted gaze may be interpreted as a call for help from us. In the context of a communal relationship, an angry averted gaze may elicit just as much of a reaction from us as does an angry direct gaze and, importantly, a distinct kind of reaction. We may wish to come to the aid of the person with an angry diverted gaze; we may still wish to protect ourselves when we perceived an angry direct gaze (suggesting our partner is mad at us) but when we care for the other’s welfare that reaction may be mixed with some feeling of responsibility for the other (especially if we did cause the anger and are in a secure, well-functioning communal relationship; cf. Yoo et al., 2011).

#### Perceiving and reacting to emotion (or events likely to have elicited emotion): data showing relational context does matter

A case for routinely taking relational context into account in interpreting studies of reactions to others’ emotional faces, and in planning for new studies also can be made on the basis results of existing neuroscience studies, that already have incorporated facets of relational context into their designs. For instance, although some have suggested that responses to self-related emotion do not overlap a great deal with responses to others’ emotion (e.g., Jackson et al., 2006), a recent study reported by Beckes et al. (2012) found that whereas threats directed at strangers produced neural responses quite distinct from those directed at the self; threats directed at *friends* produced neural responses that overlapped far more with those produced by threats directed at the self. Relationship type mattered. As Aron et al. (1991) have shown using behavioral measures, when we care about close others we often “include the other in the self” cognitively.

Work by Barrett et al. (2012) and Singer et al. (2006) also is instructive in terms of the import of relational context. Barrett et al. (2012) used fMRI to examine the effects of infant facial expressions (both positive and negative) on adults. In conducting this research they considered three of the six dimensions of relational context highlighted earlier in this article: relationship type was varied experimentally (i.e., participants viewed their own infant or someone else’s infant who was unknown to them), and aspects of relationship history (in this case participants’ history of mood and anxiety during their own postpartum period while relating to their child) as well as the participant’s own orientation toward close relationships (in this case attachment styles) were measured.

Both relationship type and participants’ histories of relationships with their own infant influenced neural reactions to pictures of infants. Regarding relationship type, participants showed greater BOLD responses in the postcentral gyrus, subgenual anterior cingulate gyrus, ventral putamen, and superior temporal gyrus in response to their own infant’s negative expression than in response to an unknown infant’s negative expression.

More interesting is another finding the authors reported. It was that that poorer postpartum quality of the participants’ maternal experience (a variable that picks up both relational history and, likely, chronic individual differences in participants’ orientation toward the relationships with their children) was significantly related to reduced amygdala response to participants’ own infants’ positive facial expressions relative to participants’ reactions an unknown infant’s positive expressions. This is a fascinating result. Perhaps all mothers, depressed and anxious or not, simply must attend to distressed infants but a history of stress, depressed moods and anxiety during the postpartum period selectively reduces some mothers’ tendencies to see their own child’s positive emotional expressions as significant. That is an important because other research suggests a child’s happiness is an important signal to caretakers (Clark and Monin, 2014), and that happiness does capture most people’s attention (Becker et al., 2011) and holds it (Power et al., 1982). Positive expressions suggest that a child is enjoying whatever is going on, thereby conveying information about what activities, foods and people a child enjoys and should be repeated or made use of when a child is not happy. The work reported by Barrett et al. (2012) suggests that all these functions of a child expressing happiness may be jeopardized among those with a history of postpartum depression, stress and anxiety. These specific results are interesting. For the present purposes, however, the overarching lesson remains that the meaning of facial expressions and neural responses to them are qualified by relational context.

Consider also the implications of the Barrett et al. (2012) findings for interpreting the results another recent study reported by Montoya et al. (2012). Montoya et al. (2012) collected neural scans that suggested that adults (in this case non-parents) experience happy infant faces as rewarding. Perhaps we are just generally built to find such faces rewarding but Barrett et al.’s (2012) results show us that in the situation in which such responses are surely most important (reacting to our *own* happy infant) that a poor relational history may cause this to go awry.

Research by Singer et al. (2006) also demonstrates that relational context matters to people’s perceptions of others’ emotional states. These researchers were interested in how research participants would react to watching a confederate in a laboratory who is experiencing pain and presumably distress. Others have examined this as well but Singer et al. (2006) added a twist to the study by *experimentally* varying relational history. Specifically, prior to viewing the confederate experiencing pain the experimenter randomly assigned half the participants to be treated fairly by the confederate in an economic game and half to be treated unfairly. That manipulated relationship history made a difference. Both male and female participants exhibited empathy-related activation in pain-related areas (fronto-insular and anterior cingulate cortices) when seeing previously fair players experience pain. However, when viewing the unfair confederate in pain and, presumably, distress, these responses were significantly lower among the male (but not the female) participants. Not only that, these males also showed increased activation in *reward* related areas when seeing the previously unfair confederate in pain and this activation correlated with these male participants’ expressed desire for revenge. Relationship history – in this case, very recent relationship history – mattered a lot for these males.

Also worthy of note is a study by Vrticka et al. (2009). It too demonstrates that relational history can be manipulated and that people’s histories of interactions with others can influence how they perceive faces going forward. These researchers manipulated people’s exposure to smiling or angry faces in the context of a game. Later they exposed the same people to these faces (now with neutral expressions) along with other neutral faces while using fMRI to record responses. As one might now expect, relational history made a difference. The results revealed that regions involved in recognizing familiar faces – the fusiform cortex, posterior cingulate gyrus, and amygdala, as well as motivational control areas such as the caudate and anterior cingulate cortex (ACC), were differentially modulated as a function of whether prior encounters with the face had been in a friendly versus unfriendly context. These results illustrate the impact of relational history well, and also show that it can be effectively experimentally manipulated.

Perhaps the most frequent way that relational context has been taken into account in this area of neuroscience research is by measuring individual differences in orientations to relationships in studies, and the most commonly examined individual differences are attachment styles. In this regard, we would simply note that when these measures have been added to several studies of reactions to others’ facial expressions, they have been shown to make a difference in the neural activity observed (see Vrticka et al., 2008; Suslow et al., 2009 for a few examples).

### GIVING SOCIAL SUPPORT

Psychologists – particularly social psychologists, but also sociologists, anthropologists, developmental psychologists, and economists – have long studied helping and other forms of what is commonly called prosocial behavior (Dovidio et al., 2006; Schroeder and Graziano, in press). More recently, a large neuroscientific literature has emerged on this topic. There are, for instance, studies using fMRI that have focused on identifying the neural correlates of reactions to signs of others’ needs (e.g., reactions to a picture of a sad other for instance; Kim et al., 2009). There also has been research aimed at eliciting actual prosocial behavior, so as to identify the conditions under which is it likely (and unlikely) to occur (e.g., Kosfeld et al., 2005; Zak et al., 2007; Izuma et al., 2010). As is the case for many studies of reactions to others’ emotions, the neuroscientific measures in much of this work (including all studies cited in the paragraph above) also have involved the collection of people’s reactions to strangers whom participants have never seen before and likely never expect to see again. Further, it is quite common for researchers to select their stimuli from one of just a few standardized sets of stimuli and these stimuli often depict stereotypical expressions that may not adequately capture the nature of facial expressions that occur in normal, everyday, social interactions^3^.

#### Indirect evidence that relational context matters

We know from a now large behavioral research literature, that relational context plays a huge role in the degree to which we respond (or fail to respond to) other people’s needs as well as in how we respond to others’ needs (Clark and Aragon, 2013). We also know that relational context matters in terms of what elicits responsiveness, in the nature of responses that are elicited, and in how people act, after having provided support to another person (see Clark and Aragon, 2013 a review). People give more support to kin than to non-kin (Segal, 1984; Essock-Vitale and McGuire, 1985; Borgida et al., 1992; Burnstein, 2005), especially when support is needed in life-threatening situations (Burnstein et al., 1994). They also give more support to those with whom they desire an ongoing relationship than to others (Clark et al., 1987). Motivations for giving support also vary – sometimes liking drives support giving; sometimes duty does so. Evolutionarily determined forces seem to drive some support giving; desire to establish business-like ties drives other forms of support giving (see Clark et al., in press). Relationship stage matters as well. We tend to give more support than we ask for as we strive to form desired relationships. In established relationships giving and seeking support tends to even out (Beck and Clark, 2009). This suggests that very early on in voluntary relationships – e.g., in friendships – help is, perhaps, given for selfish, strategic reasons; later on, it may be motivated primarily on the basis of partner need. The consequences of support giving also vary considerably. Sometimes we feel good about having helped (when it promotes relationships that are desired); sometimes we regret or feel gullible for having helped for instance when we help someone who is not special to us in any way (Williamson and Clark, 1989, 1992).

Individual differences in relationship orientations between people also matter a great deal. A striking example of this comes from research reported by Simpson et al. (1992). These scholars found that a person’s response to a close other’s needs depended on that person’s own attachment style. Those who were low in avoidance reacted to partner anxiety as one might expect and as is socially functional. That is, the more anxious their partners were, the more support they offered. However, those high in avoidance reacted in an entirely different manner. The more anxious their partners, the *less* support they provided.

All this means that, in interpreting neural correlates of giving support we should carefully consider in what relational context the data have been collected and, consequently, what their limitations might be. It also recommends intentionally including relational context in the design of neuroscience studies.

#### Direct evidence that relational context matters

Indeed, when neuroscience research *has* included relational context in designs, it typically matters. Consider some results recently reported by Telzer et al. (2011). These researchers scanned people’s brains while the people were choosing to give or not to give monetary rewards to family members. They found that decisions to give family members money were linked with activation in areas of the brain associated with self-control and mentalizing. More importantly for our point they also found that quality of the family relationships, or, in the terms we used earlier in this article, the relational character of the family relationships, mattered. Individuals who felt greater obligation to family members also showed greater functional coupling between regions related to self-control and those related to mentalizing within the ventral striatum, an area that, the authors report, is involved in reward processing. This suggests that it may well be effortful to support family members but that so doing is also rewarding for those who feel strong obligations to those family members.

For the present purposes the details of studies such as those reported by Telzer et al. (2011) and other studies in which relational context has been incorporated as a variable (see, for instance, Kim et al., 2011; Musser et al., 2012; Seifritz et al., 2013) are less important than are the overarching lessons taught by this research. Variations in relational context often will be associated with variations in the patterning of neural responses to partner needs and to actual responsiveness to partners. Moreover, only by combining knowledge about support giving, relational context and neutral responses may we come to understand just what neural responses to partner distress really mean.

Consider also lessons learned about the importance of relational context from studies on the neuropeptide oxytocin. Studies to date on the effects of experimentally varying intranasal exposure to oxytocin provide one of the most striking examples of the importance of taking relational context into account in neuroscientific studies of support giving. Early work on exposure to oxytocin and prosocial behavior involved the administration of oxytocin (or not) to individuals who were strangers to one another. Intriguing and dramatic effects emerged. One study utilized the now common ultimatum game paradigm in which one person is given money and then offers to split it any way he or she chooses between the self and the partner. Then the partner chooses to accept the split – in which case each person gets what was offered – or to refuse it, in which case each person gets nothing. In this study, participants who had been administered oxytocin intra-nasally made more generous proposals to “partners” (strangers) than did those who had not been exposed to oxytocin (Kosfeld et al., 2005; Zak et al., 2007). Some follow-up studies yielded similar effects (e.g., Israel et al., 2012). The initial study appeared in a prominent journal and received much media attention. Indeed, one author gave a widely shared TED talk (Zak, 2011) in which he declared oxytocin to be the “love hormone” and then wrote a popular book advocating a similar view (Zak, 2012). Subsequent papers urging therapeutic usage of oxytocin quickly appeared (cf. Striepens et al., 2011). Although many researchers were cautious in claims made, as just noted other researchers and many members of the media were not.

The problem was (and is) that if one-steps back and considers the literature more broadly, oxytocin does *not* always increase prosocial behavior. Indeed, not infrequently, it decreases prosocial behavior (Radke and de Bruijn, 2012 and see Bartz et al., 2011 for a review of literature on this topic). About 20% of the time intranasal administration of oxytocin actually seems to promote antisocial behavior (Bartz et al., 2011).

What predicts when oxytocin increases prosocial behavior and when it does not? The Bartz et al.’s (2011) review makes it clear that relational context is key. Intra-nasal administration of oxytocin seems to promote trust in and prosocial behavior toward benign strangers (Kosfeld et al., 2005) and toward liked others generally but *not* to outgroup members (De Dreu et al., 2011) or people suspected of being outgroup members (Radke and de Bruijn, 2012) or, necessarily, among people generally low in trust of others (Bartz et al., 2011). In other words, relational context (in this literature captured by variation in relationship types and by individual differences in people’s orientations toward relationships) can flip the effects of oxytocin on participants’ behavior changing them from pro- to anti-social in nature.

Genes also relate to variability in who does and does not respond to oxytocin with increased prosocial behavior (e.g., Poulin et al., 2012). A nucleotide polymorphism involving a guanine (G) to adenine (A) substitution is linked to sensitivity to relationship context. People homozygous for the G allele (or, sometimes, having at least one copy of the G allele) seem to benefit more from partners’ positive emotions and caring for them than those lacking G alleles (cf. Bakermans-Kranenburg and Van Ijzendoorn, 2008; Rodrigues et al., 2009) *and also* to be harmed more by a negative social context (e.g., abuse by others in early childhood (cf. Bradley et al., 2011) than those lacking G alleles. Although more research is needed, again, relational context, this time in the form of the relational character/relational histories in interaction with individual differences (here in genes), seem to matter a lot.

Importantly evidence that relational context makes a difference to the effects of oxytocin on social behavior spurred theorists to move away from declarations that oxytocin increases trust in and generosity toward others *per se*, toward more carefully considered explanations of just what the function and effects of oxytocin are. Perhaps, researchers now suggest, oxytocin increases the salience of social stimuli resulting in more positive reactions to safe, trusted, liked, or smiling people and more negative reactions to distrusted, disliked, or angry people (Rimmele et al., 2009; Sharnay-Tsoory et al., 2009). Alternatively, perhaps oxytocin increases approach tendencies (in both positive and negative ways) and damps down avoidant tendencies (Kemp and Guastella, 2011). No matter what the explanation turns out to be, it was the variability of results across relational contexts that forced theorists in this area to think about the oxytocin in a more nuanced way and that has already (and will continue) to result in better theory.

Once again, we would say: to understand neural correlates of prosocial behavior one must consider relational context. To build a good science of prosocial responding researchers must also combine the now large and rapidly expanding behavioral findings in this field by social psychologists, health psychologists, and developmentalists that takes relational context into account with neuroscience studies that also take relational context into account. It is happening to some extent in the literature; it needs to happen more and in more sophisticated ways.

### RECEIVING SOCIAL SUPPORT

People not only behave prosocially toward others; they receive support or are the target of other prosocial acts from others most typically, in daily life, in the context of friendships, romantic relationships, and family relationships. As with the topics we have covered already, reactions to receiving various forms of prosocial behavior have begun to be studied by neuroscientists, often outside the context of ongoing relationships. Examples include two recent studies, one on the impact of receiving an apology (Strang et al., 2012) and another on the impact of receiving supportive text messages after having suffered exclusion (Onoda et al., 2009). Occasionally relational context has been taken into account in such work (see Coan et al., 2006 for an example of a study in which relational context was taken into account).

Can we generalize the results of studies of neural responses to support received from strangers to what is likely to happen when support is received from well known others? We suspect not. Consider Onoda et al.’s (2009) study of having received supportive text messages from a stranger after having been rejected by a different group of strangers. These authors adopted a manipulation of social exclusion used by many previous researchers (Williams and Jarvis, 2006). That is, participants played a game of cyberball with two other players. They were included in the ball tosses at first. Later they were excluded. The exclusion elicited activity in the ACC as has been found in other studies. Later they received emotionally supportive text messages. Those whose reported social pain was reduced also showed lowered ventral ACC activation and heightened left lateral prefrontal cortex activation. The authors suggest that for these people social support enhanced prefrontal cortex activity, which, in turn, dampened activity within the ventral ACC. But when and for whom will rejection be most and least painful? When and for whom will supportive messages be most and least helpful? The answers certainly depend in important ways upon relational context. Thus, can we safely generalize results such as those of Williams and Jarvis (2006)? Probably not.

One neuroscience study of support already provides proof that relational context can shape neural responses to receiving support (Coan et al., 2006 and see also Coan et al., 2013b for a follow-up on the original study). In these researchers’ experiment, married women were told that they were in a study involving receiving shocks. On some trials they would be safe from shocks on other trials they might receive them. This occurred while participants were in an fMRI scanner. The experimenters varied whether the women received support in the form of handholding or not. Relational context also was varied in two ways. First relationship type was varied. Sometimes participants held a stranger’s hand, sometimes they held their husband’s hand, and sometimes they held no one’s hand. In addition the relational character of participants’ marriages was measured prior to the scanning session. In particular the women filled out measures of marital quality tapping satisfaction cohesion, consensus, and affection in their marriages.

Both relationship type and relational character moderated the women’s neural responses to the prosocial behavior of someone holding their hand while they were under stress. Activation in the neural systems known to underlie emotional and behavioral threat responses was most attenuated when the women held their husbands’ hands. A similar but less attenuated neural response was observed when they held the hand of a stranger compared to holding no one’s hand. In addition, among those whose husbands held their hand, the higher the quality of the woman’s marriage to her husband the less these neural areas were activated.

The behavioral literature on reactions to receiving support is long standing and fits well with Coan et al. (2006) and Coan et al. (2013b) findings. Beyond that it clearly shows that it is not just relationship type and quality that matter to people’s reactions to receiving support. People welcome non-contingent social support when they are open to a close relationship with the support giver but prefer contingent support when we prefer a more formal business like relationship (Clark and Mills, 1979). In addition, if people are secure, they seek support when it is needed; but if they are avoidantly attached they retreat when they most need the support (Simpson et al., 1992). If they are secure they perceive support as having been given voluntarily; if they are avoidant, they seem biased to see support received as having been involuntarily given (Clark and Beck, 2011). If they want to be responsive to another they are more likely to see others as more responsive (holding objective responsiveness constant) than if they do not have that desire (Lemay et al., 2007; Lemay and Clark, 2008) and the list could go on.

## RELATIONAL CONTEXT IS LIKELY TO PROVE IMPORTANT IN MANY ADDITIONAL DOMAINS

In this paper, we have chosen to emphasize the importance of taking relational context into account by focusing on research in the topical areas of perceiving and reacting to others’ emotions, giving social support, and receiving social support. These are among the most obvious substantive research areas for which relational context should matter. Yet it is important to note that the extant behavioral literature reveals that relational context can influence many, many types of thoughts, feelings, and behaviors, even ones that do not seem very social at all. Consider perceptions of the nature of one’s physical environment, for instance. Schnall et al. (2008) conducted two studies in which relational context was found to influence how steep perceivers judged a hill to be. In a first study people who judged the steepness of a hill when with a friend judged the hill to be less steep than people asked to judge the same hill in the absence of a friend. In a second study participants were assigned to think about a supportive friend, a neutral person or a disliked person and then to judge the steepness of a hill. The hill was judged to be less steep after thinking about a friend than after thinking about a neutral or disliked other. Here relational context as indexed by relationship type mattered.

Alternatively, consider ability to perform a non-social task. Woolley et al. (2010) asked groups of people to solve tasks. The average individual IQ of group members and the highest IQ of any group member positively predicted performance, but weakly. However two relational context measures were substantially better positive predictors: the group members’ ability to read one another’s emotions [as indexed by Baron-Cohen et al.’s (2001) “reading the mind in the eyes” task and how evenly distributed participation in the task was (as indexed by the group members’ turn-taking)]^4^. Here relational context as captured by individual differences in relational skills (in reading the mind in the eyes) and the relational character of groups (how evenly they shared tasks) influenced the groups’ problem solving abilities.

The point of this brief section is simple. Relational context may influence outcomes (including outcomes assessed by neuroscientists) in many, many types of tasks including ones that may not appear to be social in nature. Thus, whereas taking relational context into account in neuroscience studies that are very clearly social in nature may be especially important, so too may taking it into account more broadly in neuroscience (and in other types of research as well) prove fruitful.

## CONCLUSION

The overall points of this paper are simple: by definition *social* thoughts, feelings, and behaviors involve other people. All social thoughts, feelings, and behaviors occur within the context of a relationship with another person (even if the relationship is one between strangers interacting with one another for the first time who never expect to see one another again.) Much behavioral social, developmental and clinical work and a growing body contemporary social neuroscience research provide evidence that the relational context within which people interact (together and in interaction with other variables) is a powerful factor when it comes to shaping social feelings, thoughts, and behaviors. Yet many neuroscientists (and many other types of researchers alike, including, somewhat surprisingly, social psychologists) still study humans in isolation or, at best, when they are interacting with strangers. Often the “other people” are simply pictures of strangers. Even when people are studied while actively interacting with others and even when those others are in an ongoing relationship with the participant, often the nature of the relational context is not varied within the study nor are results of studies conducted in such relational contexts compared across studies in which the same processes were observed in other relational contexts with the explicit goal of considering how relational context may have shaped the results. It is not sufficient simply to move toward studying effects in richer more naturalistic contexts. Relational context must be considered a variable that may (and often does) shape people’s cognitions, emotions and behaviors.

If researchers are to build a coherent body of scientific knowledge about such things as empathy, support giving, support receipt (and the list could go on to cover many other topics), they must attend to relational context (including types of relationships, the character of relationships, individual differences in orientation toward relationships, histories and anticipated futures of relationships, relationship stage and where relationships sit within broader networks of other relationships). In so doing neuroscientists (and others) would be well advised to utilize, build upon and contribute to the now substantial relationship science literature. Researchers who have produced that literature have developed theory and solid empirical bodies of research characterizing these contexts in conceptual terms (Clark and Lemay, 2010; Simpson and Campbell, 2013).

Taking this literature into account is necessary to build a solid, generalizable, and integrated body of social neuroscience. In making this point we should (and do) acknowledge that, before us and continuing to the present, two other social psychologists, Ellen Berscheid and Harry Reis have urged the entire field of psychology to take relationship context into account in establishing and in integrating psychological knowledge (Berscheid and Reis, 1998; Reis et al., 2000; Reis, 2006, 2010). They have used somewhat different terms and arguments than we do here but, essentially conveying the same message. Moreover, others such as Guroglu et al. (2009) have called for studying neural correlates of social behaviors in relational context (in their case they specifically urge researchers to study these behaviors longitudinally across development as individuals acquire crucial social decision making skills). Still, this point is neglected sufficiently often that we feel it is well worthwhile to note the still largely individualistic nature of psychology studies generally (including neuroscientific studies) and to call for greater consideration of relational context.

Most recently, as we were concluding preparation of this manuscript for *Frontiers*, we read two other papers whose authors join us in making a call for more attention to relational context in research. First, Beckes and Coan (2013) published a review article on social neuroscience findings relevant to relationships in *The Oxford Handbook on Close Relationships* (Simpson and Campbell, 2013). Within this paper they too call for integrating knowledge of relationships into social neuroscience. So too do Schilbach et al. (2013) call for more neuroscience work done in social context^5^.

We are delighted to have company. We endorse several of Beckes and Coan’s (2013) suggestions for future efforts in this regard, namely that researchers should: (a) utilize relational context and, in particular “move toward the measurement of a larger variety of emotional and cognitive tasks in a relational context” because “many processes may diverge from current findings once they are tested in the presence of a loved one.” (Beckes and Coan, 2013, p. 705), (b) realize that relationship processes unfold across time and consider conducting longitudinal research, and (c) realize that taking relational context into account will enhance the chances of social neuroscience contributing to the development of clinical interventions emerging from our work.

In concluding this paper, we would re-emphasize two additional points. Social neuroscientists and other researchers alike should not be content to think of relational context just in terms of relationship types identified in lay language terms (e.g., non-parents vs. parents, or loved ones versus non-loved ones). Neither will it be sufficient to think of relational context in terms of just one type of individual difference in people’s approaches to relationships (e.g., attachment styles). We should be thinking of relationship context in clearly laid out conceptual terms and consideration should be given relational context in all its complexity including not just relationship type but also relationship character, relationship histories, relationship stages (this is *why* longitudinal studies are needed), as well as the placement of particular relationships in the context of other relationships.

Many of the conceptual variables that will prove central to our research (for instance, the trust between two people) will vary within relationships (as relational character), between relationship types, between individuals with different relationship orientations, with relational history and with the placement of relationships in larger networks of relationships. Thinking of how any construct in which a researcher is interested varies in each of these ways will allow that researcher to design studies that test hypotheses in different and more sophisticated ways and, in turn, to build a better and more integrated sets of findings.

As stated at the start of this manuscript, if social neuroscientists ignore relational context they risk establishing a body of social neuroscience that is narrow, of limited generalizability, and confusing. If relational context is taken into account as researchers build a social neuroscience of relationships, they stand a better chance of producing a coherent, integrated body of knowledge that will be intrinsically and practically valuable.

## Conflict of Interest Statement

The authors declare that the research was conducted in the absence of any commercial or financial relationships that could be construed as a potential conflict of interest.

## Footnotes

1 Social neuroscientists more frequently have taken this aspect of relational context into account than other aspects of relational context (see, for instance, Coan, 2008; Vrticka et al., 2012), but attending to this one aspect of relational context cannot completely substitute for attending to the other aspects as well.

2 There is overlap in the history of a relationship and the developmental stage of a relationship, of course. Still, there is value in considering them separately.

3 We certainly recognize the value of standardizing stimuli within studies for purposes of experimental control and across studies for purposes of being able to compare results. Our point is simply that results from studies utilizing only strangers as stimuli and only stereotypical expressions run the risk of not being very generalizable.

4 Performance also was better when more females participated.

5 We thank Reviewer 2 (Ivana Konvalinka) of this manuscript for calling this article to our attention.
